# The Elephant in the Room: A Systematic Review of Stimulus Control in Neuro-Measurement Studies on Figurative Language Processing

**DOI:** 10.3389/fnhum.2021.791374

**Published:** 2022-01-21

**Authors:** Sina Koller, Nadine Müller, Christina Kauschke

**Affiliations:** Department of German Studies and Arts, Institute of German Linguistics, Philipps University of Marburg, Marburg, Germany

**Keywords:** review, stimulus control, neuro-imaging, neurolinguistics, figurative language, idiom, metaphor, research comparability

## Abstract

The processing of metaphors and idioms has been the subject of neuroscientific research for several decades. However, results are often contradictory, which can be traced back to inconsistent terminology and stimulus control. In this systematic review of research methods, we analyse linguistic aspects of 116 research papers which used EEG, fMRI, PET, MEG, or NIRS to investigate the neural processing of the two figurative subtypes metaphor and idiom. We critically examine the theoretical foundations as well as stimulus control by performing a systematic literature synthesis according to the PRISMA guidelines. We explicitly do not analyse the findings of the studies but instead focus on four primary aspects: definitions of figurative language and its subtypes, linguistic theory behind the studies, control for factors influencing figurative language processing, and the relationship between theoretical and operational definitions. We found both a lack and a broad variety in existing definitions and operationalisation, especially in regard to familiarity and conventionality. We identify severe obstacles in the comparability and validation potential of the results of the papers in our review corpus. We propose the development of a consensus in fundamental terminology and more transparency in the reporting of stimulus design in the research on figurative language processing.

## Introduction

Our everyday language is infused with figurative expressions: when our lives turn into a roller-coaster ride, we need to keep a clear head and find a steady path again. We might even form close relationships with people with warm personalities along the way, treasuring them for their big hearts. And should we come across any more obstacles, we can take them in stride and look at the bright side of life.

The high amount of figurative expressions in language convey information on a multitude of communicative levels, e.g., affective, intentional, or simple factual messages. Therefore, the comprehension and utilisation of figurative language plays an essential role in interpersonal communication, and impairments in figurative language processing and production may lead to substantial problems with social competence (e.g., Kauschke, 2021) or mental health (Cohen et al., 2013), even though problems with figurative language can exist in the absence of any other verbal problems. Impaired figurative language processing is documented for several clinical populations that all present some kind of structural and/or functional brain deviations, such as neurodegenerative, psychiatric and neurodevelopmental disorders (Thoma and Daum, 2006) as well as patients with acquired brain trauma. Research on the structural and functional cerebral conditions of figurative language processing can therefore also serve to better understand higher order language impairments. However, results of neuro-measurement studies on figurative language processing are often contradictory due to discrepancies in definitions, terminology, and practical implementation.

A central issue in figurative language research is that there is no universally agreed upon definition of the term of “figurative language” or its subtypes, which makes it harder to pinpoint what kind of language exactly has been researched in numerous studies. In principle, figurative language represents the counterpart to literal language: the meaning intended by a speaker is not equivalent to the literal meaning of the expression. An addressee must therefore realise the inadequacy of a literal meaning to a given context, a situation or pre-existing world knowledge, making linguistic violations on a pragmatic and/or semantic level a core defining feature of figurative language (cf. Thoma and Daum, 2006).

The nature of the contrast between figurativeness and literality is a contentious point; the core issue being the question whether figurativeness and literality are inherently distinct, exclusive categories or if they represent the opposing ends of a continuum (Kasparian, 2013). On the one hand, the assumption of distinct categories carries the question of how to clearly distinguish between the two categories—where exactly lies the boundary between figurative and literal, and what exactly characterises it? On the other hand, a localisation on a continuum allows for a smooth transition between the extremes and grants a certain dynamic, potentially developmental character to any expression.

In recent decades, neuroscientific research has supplemented psychological and behavioural investigations, especially by employing neuro-imaging techniques. A key challenge of research on such a highly complex and responsive organ as the brain is stimulus control. However, the materials and methods in many studies are oftentimes not sufficently described and characterised by broad inconsistencies in definitions, terminology, and implementation, resulting in likewise inconsistent findings. The present paper aims to critically review the liguistic aspects of the research methods of functional neuro-measurement studies on figurative language, specifically on metaphor and idiom. We closely investigated the current state of research by taking a detailed look at the theoretical foundations the research papers are built upon, and by analysing stimulus design and control. As a systematic review of methods, our review explicitly does not pursue a comparison of results. We synthesised the literature to perform a quantitative and qualitative evaluation of the past and current methods with the goal of facilitating clearer, more consistent and less ambiguous research methods, enabling better comparability and validation, and advancing collective comprehension and research approaches.

## Figurative Language

Figurative language serves as an umbrella term for two main categories: phrasemes and free non-literal word compositions. Phrasemes are characterised by three main criteria: polylexicality, rigidity, and idiomacity (Burger, 2003). They are fixed, structurally non-dynamic n-grams whose meaning is not congruent with the summary of literal meanings of its constituents. The rigid sequence of words is rarely modified; exceptions may for instance occur in cases of inflection. Some cognitive models assume that phrasemes are not stored as a combination of their single components but instead as whole lexical units (Burger, 2003). Phrasemes include idioms and proverbs.

In contrast, free non-literal word compositions are not subject to rigid structure. For the subtypes of metaphor and metonymy, they constitute expressions of subconscious conceptualisation which are of a generative nature and can therefore continuously generate new expressions. In the case of the subtypes irony and sarcasm, they are utterances whose figurativeness is only spontaneously created through context and the intended evaluation of a speaker (Klappenbach and Malige-Klappenbach, 1980). The distinctions between metaphor and other figurative subtypes are not universally agreed upon, leading to varying operational definitions in empirical studies. In the following, we aim to characterise core features in order to arrive at working definitions for our review, which includes studies on metaphor and idiom processing only. These two subtypes constitute the majority of figurative language examined in neuro-scientific research, therefore representing a solid basis for our review. For an overview of working definitions of figurative language subtypes other than metaphor and idiom, see Supplementary Table S1^1^.

### Subtypes of Figurative Language: Metaphor and Idiom

#### Metaphor

Cognitive linguistics considers metaphor a cognitive means of conceptualising abstract issues by means of concrete experiences. In 1980, Lakoff and Johnson (1980) introduced their Conceptual Metaphor Theory (CMT), which will provide the terminology for the present paper.

The CMT is based on the embodiment hypothesis: “The detailed nature of our bodies, our brains, and our everyday functioning in the world structures human concepts and human reason” (Lakoff and Núñez, 2009, p. 5). This embodied experience of our bodies in a three-dimensional environment determines human learning processes which continuously build on each other; thought is therefore not inherently abstract or independent from our bodies (Kövecses, 2010).

In order to understand internal and external sensations and to be able to interact adequately with ourselves and the environment, a certain cognitive structuring mechanism is required. The stimulations we experience naturally vary in their complexity—compare, for example, the warmth and tactility of a freshly made cookie in our hand with the complexity and abstractness of a political debate.

In order to facilitate comprehension of complex and not primarily bodily grounded concepts, conceptual metaphors link these concepts with simpler, less abstract concepts and therefore act as a subconscious mechanism of conceptualising our experiences through our environment and self. In principle, a conceptual metaphor links a concrete source domain (e.g., machine) with a more abstract target domain (e.g., mind) by mapping relevant elements of the source domain onto elements of the target domain. The resulting basic form of a conceptual metaphor can therefore be phrased as “A is B” (“the mind is a machine”), where A is the target domain and B is the source domain. The conceptual metaphor, a cognitive mechanism, now generates actual expressions on the linguistic surface (“that's what makes her tick,” “the holidays allowed them to refuel,” “you can watch the wheels turning in his head”). The abstract target domain (mind) is made accessible by the tangible source domain (machines) by using the pre-existing comprehension and retrievable experiences of the source domain for conceptualisation (Lakoff and Johnson, 1980; Kövecses, 2010).

Conceptual metaphors are of a creative, generative nature: the conceptualisation mechanism can steadily give rise to new linguistic expressions. These novel metaphors can then be conventionalised through continuous usage within a language community. Bowdle and Gentner (2005) describe this potential development in their model of the Career of Metaphor: the conventionalisation of a novel metaphor is a gradual process at whose end the metaphor can even become a “dead metaphor”—a metaphor that has entirely lost its figurative character and has become lexicalized (Schmidt et al., 2009). Words like “table leg” or “laptop” are lexicalised as complete units and do not require any mappings for comprehension. Not every metaphor is subject to the career of metaphor—some are simply only conventionalised to a certain degree, many never reach this point or disappear from common language usage.

For the purposes of this review, we define metaphors as free non-literal expressions which are not subject to rigid structure and which follow the notion of conceptual metaphor after Lakoff and Johnson's CMT (Lakoff and Johnson, 1980), i.e., metaphors as a conceptualisation mechanism with a source and a target domain.

#### Idiom

Both metaphor and idiom are highly frequent in colloquial language usage (Gibbs and Beitel, 1995; Thoma and Daum, 2006) and represent a group of expressions whose figurative meaning is not composed by the literal meanings of its constituents. However, idioms are a subclass of phrasemes, i.e., they do not follow the usual linguistic-productive rules and are not a creative-generative class (Dobrovol'skij, 1995).

Idioms are not a uniform class but can instead differ in many aspects. One of those is non-compositionality, i.e., the non-additivity of the meanings of single constituents from the perspective of the total meaning of the idiom (Dobrovol'skij, 1995). The figurative meaning of semantically non-transparent/opaque idioms cannot be extracted from the literal meanings of its constituents (e.g., “kicking the bucket”); semantically transparent idioms however contain components in their literal meaning (e.g., “pouring money down the drain”; Canal et al., 2017). Some idioms can be understood through transferred metaphorical comprehension (“putting one's cards on the table,” “taking something in stride”), which reveals the possibility of overlap with highly conventionalised metaphors. Another dimension characterising idioms is the degree of their literal interpretability (literality). Idioms such as “being on thin ice” or “a piece of cake” do allow for a literal interpretation, although it will seem unsuitable in most contexts. Idioms such as “the elephant in the room” or “raining cats and dogs” however refer to unrealistic or entirely impossible scenarios, giving stronger indication for an intended non-literal meaning.

The two primary dimensions characterising idioms and distinguishing them from metaphors, however, are syntactic stability and conventionality. Idioms are generally considered conventionalised (Desai et al., 2013; Canal et al., 2017); some authors go as far as equating idioms with dead metaphors (Mashal et al., 2014) or describing metaphors as a subgroup of idioms (Rapp and Wild, 2011). However, idioms do not necessarily have to be of a metaphorical nature. Idioms also generally possess the rigid syntactic structure of phrasemes: they are highly collocating n-grams. It is often argued that their meaning is learned as a whole and stored as a unit in the mental lexicon (Gibbs and Beitel, 1995); other approaches however propose different models (cf. Mashal et al., 2014; Canal et al., 2017). Consequently, we use the following working definition: idioms are conventional multi-word expressions of rigid syntactic structures whose meaning cannot be extracted by the meaning of its single constituents.

### Neural Processing of Figurative Language

The specifics of the neural processes underlying figurative language processing are the subject of considerable debate (cf. Thoma and Daum, 2006; Bohrn et al., 2012; Kasparian, 2013; Wang and He, 2013; Diaz and Eppes, 2018). A primary issue in the research on the cerebral localisation of figurative language processing is the specialisation of the hemispheres, and research on finer localisation has supplemented this focus. Functional neuro-measurement methods are able to visually display cerebral processes and allow for the neural investigation of online language processing, i.e., the processing of language at the point of measuring. Our review includes studies using functional magnetic resonance imaging (fMRI), electroencephalography (EEG), positron emission tomography (PET), magnetoencephalography (MEG), and near-infrared spectroscopy (NIRS).

Generally, in right-handed people the left hemisphere (LH) has been proven dominant for basic language processing but early studies reported a critical role of the right hemisphere (RH) for the understanding of metaphors (Winner and Gardner, 1977). The hypothesis of a special role of the RH was reinforced in the 1990s by Bottini et al. (1994) and by a divided visual field (DVF) study by Anaki et al. (1998). Both studies tested neurologically healthy participants and observed a dominance of the RH in the processing of metaphors. On the other hand, other studies could not find any special involvement of the RH (e.g., Rapp et al., 2004; Lee and Dapretto, 2006; Stringaris et al., 2007). A comparison of studies representing opposite hypotheses on the involvement of the RH reveals a fundamental problem: most of these studies are so different in their design, their material, and their execution that a general comparison is hardly possible (see below, also cf. Thoma and Daum, 2006; Bohrn et al., 2012; Kasparian, 2013).

Some models attempt to explain the potential lateralisation differences between literal and figurative language. The two most prominent among these models are the graded salience hypothesis (GSH, Giora, 1997) and the coarse semantic coding theory (CSCT, Beeman et al., 1994; Jung-Beeman, 2005). Both approaches share the assumption that it is not figurativeness itself but instead other characteristics that are the cause for hemisphere specialisation.

Giora's GSH considers salience the critical factor for hemispheric differences. Giora defines salience as a combination of familiarity, conventionality, frequency, and predictability of the meaning of an expression. Processing is therefore not determined by an objective contrast between literal and figurative but depends on the subjective context and previous contact with possible meanings. According to this hypothesis, the LH is responsible for the processing of salient meanings, while the RH is called upon for the processing of non-salient meanings. The figurative meaning of dead metaphors or already familiar idioms would be salient, the metaphorical meanings of unfamiliar metaphors would be non-salient.

The CSCT is based on the semantic-lexical network of an individual speaker: the theory attributes the responsibility of fine semantic coding to the LH, i.e., the activation of closely related word meanings and semantic features. The RH, on the other hand, activates weaker, more diffuse, big semantic fields and is therefore involved in the processing of ambiguities, synonyms, and more broadly related meanings. Since the meaning of figurative expressions, especially metaphors, is often semantically more distant than the literal meaning, these more broadly activated semantic fields are necessary—the semantic fields of single words of polylexical expressions overlap at critical points for relevant mappings, enabling the comprehension of the figurative meaning. Consequently, this increasingly recruits the RH for figurative language whose meaning is not part of the close semantic environment of its single constituents. Both approaches therefore agree that not all figurative expressions can be treated as a uniform collective, but instead have to be more finely distinguished. Furthermore, both models emphasise the subjectivity of language experience and the importance of controlling for possible influence factors.

Research on activation localisation is not only limited to the role of the hemispheres but also examines finer areas. In 2012, Bohrn et al. (2012) conducted a meta-analysis in which they collectively analysed the data of studies concerned with online figurative language processing. A predominant area proved to be the left inferior frontal gyrus (IFG) when contrasting figurative with literal language; the IFG appears to be more strongly involved in metaphor and idiom processing than in irony or sarcasm. Overall, a picture of a bilateral network with a dominance in the LH emerges: the bilateral IFG, temporal lobe, medial frontal gyrus and left amygdala show increased activation in the processing of figurative language (Bohrn et al., 2012). Bambini et al. (2011) also describe a bilateral network that includes the left angular gyrus and the anterior cingulum in addition to the bilateral IFG and superior temporal gyri. It is important to note that special activation for literal language but not for figurative language is reported in only about a third of the studies examined by Bohrn et al. (2012). This may point toward the processing of figurative language generally using the same network as the processing of literal language, but requiring additional cognitive resources. The cognitive load in language processing does not only depend on the distinction between figurative and literal, but is also influenced by a number of factors characterising the stimulus material.

### Factors Influencing Figurative Language Processing

The successful processing of figurative language requires the integration of cognitive, affective, communicative, social, and linguistic information (Farnia, 2018). Our review will examine in detail how relevant studies control their stimuli for (psycho-)linguistic factors empirically. For this purpose, we will give our working definitions for the most prevalent influence factors that were shown to influence the neural response in figurative language research. In a first step, we collected all influence factors mentioned in several reviews on figurative language processing (Blasko and Connine, 1993; Thoma and Daum, 2006; Rapp and Wild, 2011; Bohrn et al., 2012; Rapp et al., 2012; Vartanian, 2012; Kasparian, 2013; Wang and He, 2013; Lundgren and Brownell, 2016; Diaz and Eppes, 2018). During the further literature analysis, the list was inductively extended by other factors frequently controlled for. All of the following influence factors were included as analysis factors in our review, serving as indicators for the depth, scope and implementation of stimulus control. We divided the influence factors into two categories: pycholinguistic factors, e.g., psycholinguistic variables, whose values are dependent on personal (linguistic) experience, and structural factors, e.g., syntactic complexity or length, which are intrinsic characteristics of linguistic stimuli and not dependent on individuals' perspectives.

#### Psycholinguistic Influence Factors

##### Valence

Emotional valence measures how pleasant (or positive) or unpleasant (or negative) a linguistic expression is perceived to be (Russell and Barrett, 1999). It therefore represents one part of affect, the conveyance of which is an important function of figurative language (Cardillo et al., 2012). Highly emotionally valenced words have been found to be processed with priority (especially positively valenced words, resulting in a “positivity superiority effect,” Lüdtke and Jacobs, 2015) and to elicit stronger event related potential (ERP) components associated with emotional processing (cf. Citron et al., 2016a). Differently valenced expressions have also been shown to result in different activation patters in both children and adults (Sylvester et al., 2021) and several studies found metaphors to be more emotional than literal expressions (Gibbs, 2002; Citron and Goldberg, 2014; Mohammad et al., 2016).

##### Arousal

Arousal joins the factor of valence as the second factor of affect. It measures the physiological activation caused by a stimulus, i.e., how “exciting” the stimulus is (Russell and Barrett, 1999). A verbal expression is therefore localised on two axes indicating its affectivity: valence encompasses negative and positive experience, while arousal indicates how stimulating, or intense, an expression is (Jacobs et al., 2015). Both factors have been found to behaviourally and neurally influence (figurative) language processing, specifically word processing (Kuperman et al., 2014; Kever et al., 2019; Pauligk et al., 2019).

##### Familiarity

Idioms, proverbs, and metaphors can be known or unknown to speakers—this subjective previous experience with figurative expressions is called familiarity (Schweigert, 1986; Titone and Connine, 1994). Familiarity is a crucial influence factor; the more experience a speaker has with a figurative expression, i.e., the more they hear it, read it, or use it themselves, the deeper it ingrains itself in their language usage and is integrated into the close semantic field of the single components. Given this close semantic relationship within one expression and the increased salience of familiar items according to the GSH, familiar figurative expressions are indicated to be processed more efficiently and directly than unfamiliar ones (cf. Schmidt and Seger, 2009). The term familiarity is often used synonymously with the term conventionality in the literature.

##### Conventionality

On the surface, conventionality may easily seem synonymous with familiarity as both terms refer to a certain degree of usualness. However, the two terms have to be distinguished clearly. Conventionality refers to the entrenchment of a figurative expression (proverb, idiom, metaphor) in the collective general language usage (Lai et al., 2009), which is enabled by frequent use by a significant number of speakers of a language community (Forgács et al., 2012; Goldstein et al., 2012). Consequently, conventionality does not carry an individual-subjective component but instead refers to the familiarity with an expression on the level of a speaker collective. To illustrate, consider non-native speakers: the German idiom *jemanden auf den Arm nehmen* (literally: “take somebody onto the arm,” meaning “to kid,” “to tease”) is conventional in German language usage but an English speaker learning German has not yet encountered the expression often enough (or at all) to become familiar with it. The learner has therefore now been inducted into a language community where the idiom is conventional, but it does not possess any individual familiarity for them. Citron et al. (2020b) indeed reported processing differences of conventional metaphors for L1 and L2 speakers, demonstrating the need for a careful distinction between individual familiarity and collective conventionality.

##### Frequency

The frequency of proverbs, idioms, and metaphors strongly correlates with familiarity; the more frequent an expression, the more familiar speakers tend to be with it (Rapp, 2005; Tanaka-Ishii and Terada, 2011). Per definition, the frequency of metaphorical meanings cannot be measured objectively, which is why alternative means have to be found. Frequency has been considered the frequency of occurrence in corpora, taken from normed databases or been rated subjectively; one must also distinguish between the frequency of entire polylexical compositions and the frequency of single words. Depending on the method of measurement, frequency has been used interchangeably with familiarity and conventionality (cf. Kasparian, 2013), leading to confounding of the respective factors. We use frequency of occurrence as our working definition.

##### Concreteness/Abstractness

The definition of concreteness is subject of dispute, as well. Forgács et al. (2015) equate the term “concrete” to “physical”; “abstract” consequently means “not physical” here. Citron et al. (2016b) however describe concreteness as referring to “a state or event that one can experience in one or more sensory modalities”; abstract things are therefore not tactile, audible, visible, smellable, or tasteable (Paivio et al., 1968). This broadens the definitions of concreteness and joins it with the theory of embodiment: the most direct experiences are those with one's own body, which then serve as reference points to abstraction. Figurative and literal expressions can markedly differ in their concreteness. For the purposes of this paper, we follow the definition by Citron et al. (2016b) and localise verbal expressions on a continuum between concrete and abstract.

##### Imageability

Imageability is linked to the factor of concreteness; the two factors are not always used in clear separation (e.g., Lachaud, 2013; Lai et al., 2015). Imageability refers to the ease with which an expression evokes a mental image. Concreteness and imageability have been shown to influence recall duration and comprehension difficulty (Barry and Gerhand, 2003; Sabsevitz et al., 2005).

##### Comprehensibility

Neuroscientific research papers use many terms to refer to the basic comprehensibility of a stimulus (e.g., Rapp et al., 2004; Mashal et al., 2005; Ahrens et al., 2007; Diaz et al., 2011; Cardillo et al., 2012; Lacey et al., 2017): understandability, comprehensibility, ease of understanding, and interpretability. These terms essentially describe how accessible and easy the comprehension of the meaning of the stimuli is. This factor naturally does not exist isolated from other characteristics of the stimuli—the activation of cognitive resources for instance depends on familiarity, syntactic complexity, and context (Schmidt and Seger, 2009).

##### Plausibility

The factor of plausibility is sometimes used in overlap with comprehensibility. However, it does not refer to the individually perceived difficulty of comprehension, but describes the degree of sensicality and therefore measures the meaningful content of linguistic stimuli (Weiland et al., 2014). For figurative expressions, one must distinguish between literal and figurative plausibility—for instance, some metaphors may be literally plausible (“an upstanding person”) but this meaning is not the intended one; other metaphors are literally implausible (“she is an angel”; Zempleni et al., 2007). The terms meaningfulness and sensicality have been used synonymously with plausibility (e.g., Stringaris et al., 2007; Weiland et al., 2014; Zane and Shafer, 2018; Jończyk et al., 2020).

##### Compositionality/Transparency

In regard to idioms, compositionality refers to the degree to which the components of an expression contribute to its total meaning (Laurent et al., 2006; Mashal et al., 2008). As detailed above, most definitions characterise idioms as non-compositional (Mashal et al., 2008; Zhang et al., 2013); however, some idioms are semantically transparent.

##### Context

The context of a linguistic expression, i.e., the linguistic (Diaz and Eppes, 2018) and situational environment, crucially determines the effort of semantic processing (Sela et al., 2015). The (in)adequacy of the literal meaning of an expression in relation to its context is an essential characteristic of figurative language: the clearer the context indicates a certain meaning, the easier the (subconscious) choice between literal and figurative interpretation. Context fulfils a disambiguating role and consequently influences the predictability of a certain meaning of an expression (Cacciari and Tabossi, 1988). The meaning of proverbs and idioms can indeed be stored independent from context; however, context can play a crucial role in these cases as well (compare the statements, “My week will be hectic because I have a lot on my plate” vs. “I knew my kid wasn't going to finish their dinner because they had a lot on their plate”). Furthermore, ironic and sarcastic meaning cannot exist independently from context.

##### Cloze Probability

Cloze probability refers to the probability of a certain word completing a certain expression given the preceding context (Lai et al., 2019): it is therefore a kind of context-dependant expected value. The CP influences essential components in EEG (Weiland et al., 2014) and can vary between literal and metaphorical expressions (Coulson and Van Petten, 2007). Context does not necessarily mean extensive context consisting of several sentences; the beginning of phrase or a sentence can suffice as a prior condition for CP.

##### Salience

This factor integrates several other factors and interacts dynamically with a given context (see above). For our review, we define salience according to Giora's GSH, i.e., a combination of familiarity, conventionality, frequency, and predictability.

##### Figurativeness

Although it may at first seem circular to mention figurativeness as an individual influence factor, one has to remember the behavioural and neural differences in the processing and production between figurative and literal language. Controlling stimuli for their actual figurativeness avoids the possibility of classifying subtly figurative stimuli as literal or vice versa.

#### Structural Influence Factors

##### Part of Speech

Linguistic stimulus material can consist of various parts of speech; it is especially important to which part of speech the critical (i.e., figurative) elements of the material belongs. In nominal metaphors (“he is a treasure”) a noun carries the figurative meaning, this function can also be conveyed by verbs (“the praise made her soar”), adjectives (“he is a broken man”) and prepositions (“she is beside herself”). Since parts of speech refer to different concepts (things/emotions/states of being vs. actions vs. relations), they entail different levels of abstraction (cf. Lai et al., 2019).

##### Tense

If stimulus material contains verbs and if these verbs are not used as isolated infinitives but instead are embedded in a phrase or a sentence, the tense of the stimuli has to be considered. Tenses have been found to be processed differently on a cerebral level (cf. Desai et al., 2006; Gilead et al., 2013) and to be conceptualised by different means in a figurative sense (cf. Gilead et al., 2013; Parkinson et al., 2014), making tense a potential confounding factor.

##### Length

Stimuli of different lengths engage the working memory to a different degree (Pointe and Engle, 1990; Tehan et al., 2001), and longer stimuli naturally require longer reading or listening times (Bonin et al., 2013). The length of linguistic stimuli can be stated in letters, phonemes, syllables, words, or entire sentences, in the case of auditory stimuli the temporal duration can be given as well. Depending on the nature of the stimuli, one unit of measurement might be more suitable than others; it is however not important which unit of measurement is used but rather that length is controlled for at all.

##### Syntactic Complexity

Not only the length of stimuli but also the actual syntactic complexity has to be considered. In the case of phrasal or sentential stimuli, stimuli can contain a broad spectrum of syntactic structures. With increasing complexity more cognitive resources are activated (Citron et al., 2016b), which in turn influences the recruitment and functional connectivity of the hemispheres (Thoma and Daum, 2006).

The influence factors mentioned above all play a role in the processing of figurative language. Since the characteristics of these factors vary between literal and figurative meanings—for example, a bitter feeling has a more negative connotation than a bitter taste—most norms for figurative language cannot be extracted from databases based on literal language alone. To obtain reliable values, they have to be rated by a large number of native speaking individuals, expending a lot of time and resources. To have all possible influence factors rated in advance of a study is therefore an unrealistic expectation. However, there are specific metaphor and idiom databases in many languages, such as English (e.g., Cardillo et al., 2010, 2017; Nordmann et al., 2014), German (e.g., Citron et al., 2016a, 2020a; Müller et al., 2021), Italian (Bambini et al., 2014), Spanish (Gavilán et al., 2021), Bulgarian (Nordmann and Jambazova, 2017), French (Bonin et al., 2013, 2018), Chinese (Li et al., 2016), and Dutch (Hubers, 2019). Figurative stimuli and scores of influence factors can be extracted from these databases and used in empirical research on figurative language.

## Methods

The aim of the present review lies in systematically investigating the theoretical background and the research methods of neuro-measurement studies on figurative language, specifically on metaphor and idiom. Our leading questions are:

(a) Definitions: How are subtypes of figurative language defined and distinguished, and which criteria mark the distinctions?

(b) Influence factors: Which stimuli characteristics are controlled for, and how are the control factors defined and implemented?

(c) Participants: Which populations are tested in the studies and what are their fundamental characteristics?

### Inclusion Criteria

Our review follows the PRISMA guidelines (Moher et al., 2009). Since the research on the comprehension of figurative language stretches across many scientific fields and makes use of a diverse number of methods, an extensive number of research papers have been published over the past decades. The present review sets the inclusion criteria stated in Table 1.

**Table 1:** Inclusion criteria for our review.

Publication medium	Empirical research papers exclusively
Language of publication	English
Language of stimuli	Any
Measuring method	Neuro-measurement methods only (EEG, fMRI, PET, MEG, or NIRS); applied during online language processing
Time of publication	1990 to 2021 (“today” at time of review)
Participants	Any, i.e., healthy and clinical populations
Figurativeness	Stimuli have to be both linguistic and figurative. This excluded studies that used figurative pictures or gestures only, or where linguistic stimuli only gained figurative meaning in association with gestures or position relative to the participants' viewpoint. Multimodal studies combining figurative linguistic material with pictures, gestures or movement were included in the review
Subtypes	Search process included all types of figurative language; review includes metaphor and idiom only

### Process

Given the above criteria, not every literature database presented a suitable source for our review. For practical reasons, we worked with databases that had to be accessible to the public or via a university account, and had to offer advanced search functions (i.e., allow for logical operators) and export functions. We therefore selected four databases: PubMed (pubmed.ncbi.nlm.nih.gov), Cochrane (www.cochranelibrary.com), Google Scholar (scholar.google.com) and Web of Science (WoS, webofknowledge.com).

By screening already available literature summarising neuroscientific research on figurative language, we inductively collected keywords that were to serve as critical search items. Those keywords fell into two categories: linguistic (figurative language, non-literal, proverb, metaphor, idiom, metonymy, simile, sarcasm, irony) and neuroscientific (neuro^*^, imaging, brain, hemisphere, fMRI, EEG, PET, ERP, MEG). For the final search term, we combined the first with the latter with an additional specification to single out papers where the linguistic keywords occurred in context of applied (neuro-)linguistics^2^. The keywords also had to occur in the title and/or abstract; full-text searches were avoided explicitly. The literature accumulation in all four databases began August 5th 2020 and ended August 10th 2020. The search was repeated, with a publication time widow set to 2020–2021, on August 30th 2021 in order to update the review corpus. (See Supplementary Material C) for an example of the full search term and restrictions.

In addition, the source material of ten already available reviews on related topics (Blasko and Connine, 1993; Thoma and Daum, 2006; Rapp and Wild, 2011; Bohrn et al., 2012; Rapp et al., 2012; Vartanian, 2012; Kasparian, 2013; Wang and He, 2013; Lundgren and Brownell, 2016; Diaz and Eppes, 2018) was systematically screened for relevant literature which was subsequently added to the database search results. All ten of these reviews had other foci than the present review. None examined the linguistic research methods in quantitative and qualitative detail, which was the purpose of our review.

The result was a raw literature corpus encompassing several hundred sources. In a next step, we manually sorted this corpus using the open source software JabRef (JabRef Development Team, 2020). After deleting all duplicates, we judged the remaining sources on their suitability based on title and abstracts, using the inclusion criteria described above and following the PRISMA process (Moher et al., 2009).

For the in-depth analysis of the final corpus of research papers (“review corpus”), we entered all relevant data into a structured database (“analysis chart”) using LibreOffice Calc (The Document Foundation, 2021). Note that an in-depth analysis was undertaken for papers on metaphor and idiom only, all other literature is merely listed as a source along with measurement method and figurative subtype, and is available for further research.

Please refer to the analysis chart (Supplementary Table S2) for a detailed description of the purposes of each analysed aspect. The complete analysis chart is available in Supplementary Material A and at https://osf.io/hpzb8/. All data was analysed with LibreOffice Calc and R (R Core Team, 2020).

## Results

### Literature Identification

**Figure 1:**
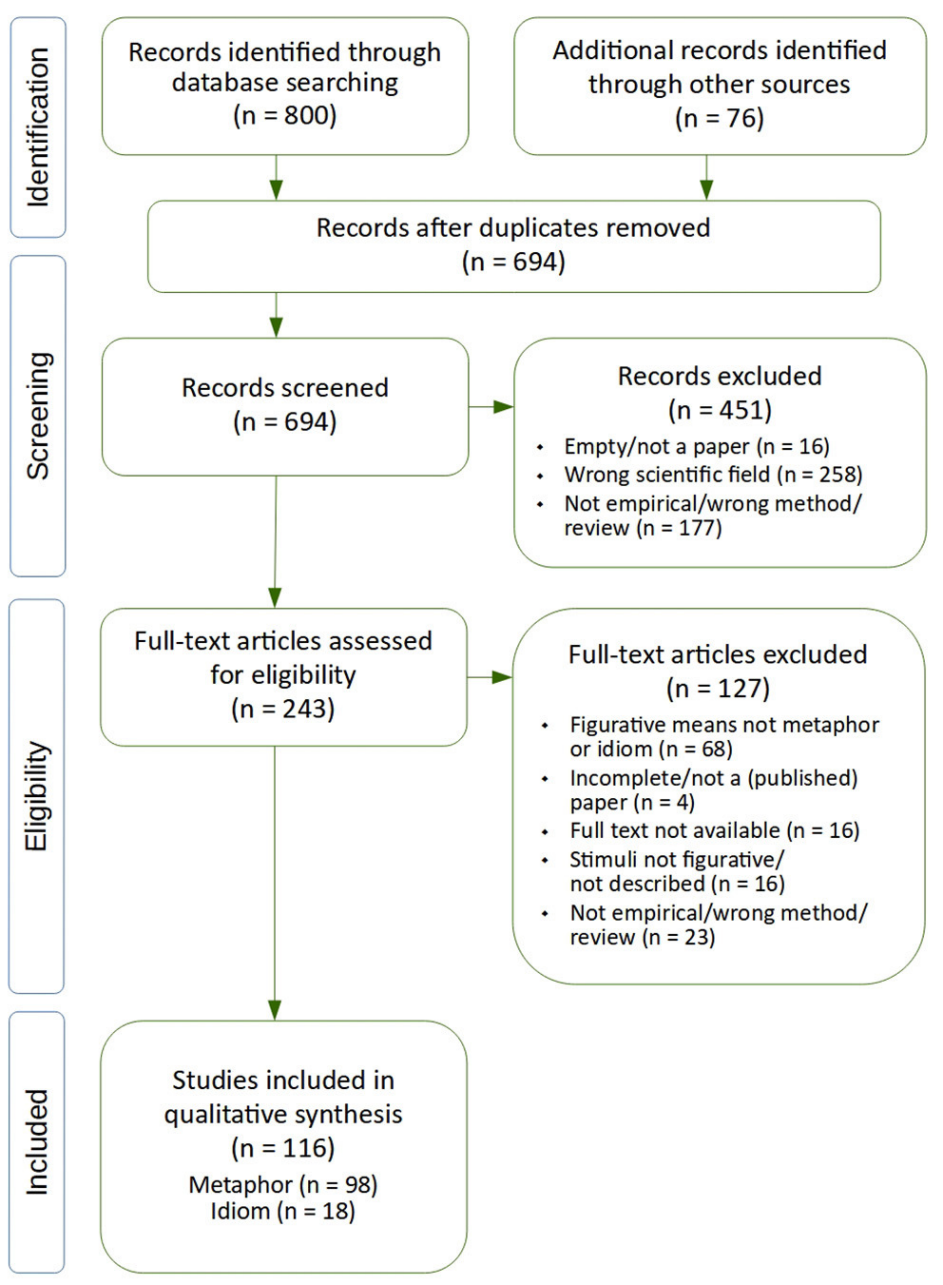
The literature synthesis process. Graphics template by Moher et al. (2009).

The literature identification resulted in 116 research papers (Supplementary Table S3) which we accepted as suitable material for our review. 98 papers claimed to have worked with metaphors, 18 described their stimuli as idioms. For details on the selection process, (see Figure 1).

The papers were published between 1994 and 2021, giving representation to 28 years of research. In regards to measurement methods, fMRI was applied most frequently. For more details, (see Supplementary Figures S1, S2).

### Definitions of Figurative Language

Figurative language as a term in itself is defined in 13 of the 116 research papers. However, we found definitions and differentiations for the subtypes more frequently included: of the 98 papers on metaphor, 50 (=51%) define the term of metaphor. Ten out of the 18 papers on idiom (=55.6%) define the term of idiom. For these numbers, we deliberately only included definitions that mentioned formal criteria and/or cognitive modalities, e.g., mappings. If a paper merely listed an example instead of including a definition, this was not counted as a viable definition.

Where included, metaphor is primarily defined in its function as a cognitive conceptualisation mechanism, mostly by way of the roles of source and target domains (or “topic” and “vehicle”) and mappings. In total, metaphor is defined in 57 papers, i.e., almost 50% of all articles included in our review. In 15 of these cases, it is distinguished against other figurative subtypes (among that idiom: *n* = 10).

Conventionality may serve as the distinguishing factor between metaphor and idiom in most cases (e.g., Laurent et al., 2006; Zempleni et al., 2007; Lauro et al., 2008; Desai et al., 2013; Mashal et al., 2013, 2014; Pomp et al., 2018). Idiom is defined in 19 out of all research papers, and other subtypes are explained only in context with metaphor and idiom (irony: *n* = 6; simile, metonymy: each *n* = 2; proverb, sarcasm, hyperbole: each *n* = 1).

In terms of actual implementation, studies on metaphor processing clearly outweigh studies on idiom processing in our review corpus. 98 papers report studies on the first, and 18 are concerned with the latter. Eleven papers contrast metaphors with another figurative subtype in their paradigms, i.e., the stimuli included figurative subtypes besides metaphor: idiom (Desai et al., 2013; Romero Lauro et al., 2013; Lorusso et al., 2015), irony (Eviatar and Just, 2006; Prat et al., 2012; Deckert et al., 2021), metonymy (Weiland et al., 2014; Yurchenko et al., 2020), sarcasm (Uchiyama et al., 2012), and simile (Shibata et al., 2012; Lai and Curran, 2013).

The majority of introductions and theoretical background sections of the papers refer to cognitive models of figurative language processing (*n* = 81). For more detail, (see Supplementary Table S4).

### Stimulus Design

We found a wide variety of factors that the studies controlled for. Table 2 summarises the numbers of papers controlling for each psycholinguistic and structural factor, calculated from a binary analysis system (controlled for/did not control for).

**Table 2:** Number of studies controlling for each influence factor. Total studies: *n* = 116.

**Control factor**	***n***	**Control factor**	***n***
Length	86	Imageability	29
Frequency	69	Cloze probability	28
Familiarity	66	Tense	27
Syntactic complexity	62	Comprehensibility	27
Other	54	Valence	25
Part of Speech	52	Conventionality	10
Figurativeness	45	Arousal	7
Concreteness	32	Compositionality/Transparency	7
Plausibility	33	Salience	2

These totals only allow for superficial insight, however. As detailed in the introduction, there are no generally accepted definitions for the pycholinguistic factors. For the purposes of the present review, we meticulously examined which definitions were mentioned and which were operationalised. Consequently, we did not indiscriminately trust the statements of the papers but instead compared the respective definitions with our working definitions as stated in the introduction. The classification in this review follows our working definitions. Thus, we occasionally classified some factors contrary to their respective papers' statements. This was the case, for example, with papers that defined conventionality as individual-subjective (e.g., Mashal et al., 2005, 2007; Lai et al., 2009; Subramaniam et al., 2012; Tang et al., 2017) and were therefore registered under “familiarity.” We proceeded similarly with papers such as Mashal et al. (2005) and Kircher et al. (2007), which claimed to have controlled for salience but only considered one aspect of salience (e.g., familiarity or frequency).

From a quantitive perspective, 64 out of the 116 (55%) research papers do not contain any definitions of their psycholinguistic influence factors, regardless of whether their definitions were congruent with our working definitions or not. Four studies (Iakimova et al., 2005; Vespignani et al., 2010; Lu and Zhang, 2012; Wang et al., 2021), i.e., 3.4%, define all factors for which their stimuli are controlled. The remaining 48 papers define at least one factor.

In the following, we will focus on the most prominent and most frequently controlled factors, representing the current status quo in stimuli control.

#### Psycholinguistic Influence Factors

Among the psycholinguistic influence factors, familiarity is the one most frequently controlled: 69 out of the 116 studies included this variable in their control processes. Furthermore, 33 papers examined either “familiar” or “conventional” metaphors according to their own statements. In 19 of those papers, the two terms are explicitly used synonymously, three out of the 18 also use them synonymously with “salience” (Ahrens et al., 2007; Mashal et al., 2007; Mashal and Faust, 2009). 15 out of the 18 papers equalling familiarity with conventionality describe a factor most similar to our working definition of familiarity. They therefore specify a subjective-individual familiarity and not the entrenchment of an expression in the general language usage.

A central term in the familiarity vs. conventionality issue is “novel”. 25 studies examine “novel” metaphors (21 of them use this term to describe their experimental conditions, in 16 studies it is part of the article title), i.e., metaphors that are “new” in some kind of way. 16 papers oppose “novel” to “conventional”, three use it as the opposite to “familiar,” and the remaining six studies use it synonymously with “non-frequent,” “poetic,” “unusual,” or give no indication of the meaning.

Furthermore, we observed a difference between the definition and operationalisation of defined terms and influence factors, for instance “novel” and familiarity. The actual realisation of the term “novel” in the relevant studies marks a stark contrast to the initial definitions given by the papers. In the majority, “novel” stimuli are operationalised as stimuli with low familiarity scores (Mashal et al., 2005, 2007, 2013, 2014; Ahrens et al., 2007; Lai et al., 2009; Yang et al., 2009, 2010; Diaz et al., 2011; Cardillo et al., 2012; Lai and Curran, 2013; Zeev-Wolf et al., 2015; Hartung et al., 2020). Only four studies can be classified as having worked with conventionality in this respect (Goldstein et al., 2012; Subramaniam et al., 2012, 2013; Jończyk et al., 2020). Three studies (Forgács et al., 2012; Schneider et al., 2014; Forgács, 2020) determine the “novelty” of their stimuli by means of frequency of occurrence in corpora or in Google searches, one adheres to their definition of “poetic origin” (Mashal and Faust, 2009) and four papers make no statement as to the actual implementation (Tartter et al., 2002; Arzouan et al., 2007a,b; Beaty et al., 2017).

In a similar vein, the factor familiarity illustrates discrepancies between definitions and actual scoring implementation. A number of studies explicitly define familiarity as a continuous variable (Diaz et al., 2011; Cardillo et al., 2012; Mashal et al., 2014; Lai et al., 2015). A higher number of studies demonstrates an implicit assumption of continuous scales for various variables by having those variables rated on multi-point scales. However, the resulting continuous scores of those ratings are then binarily operationalised by a cut-off value (Mashal et al., 2005, 2007, 2013; Arzouan et al., 2007b; Yang et al., 2010; Goldstein et al., 2012; Lorusso et al., 2015), e.g., by choosing “3” as a cut-off value between “novel” and “conventional.” Some papers do not specify any criteria for their distinctions (e.g., Diaz et al., 2011; Lai et al., 2015).

#### Structural Stimulus Design

As a consequence of the inclusion criteria, all 116 studies employed linguistic-figurative stimuli. 14 studies employed additional media (for more details, see Supplementary Figure S3).

The number of experimental stimuli varies markedly across studies, ranging from a total of 22 stimuli (Citron et al., 2016b) to a total of 1,024 (Kircher et al., 2007) with a total average of 194 stimuli. Concerning figurative stimuli only, we found a minimum of 10 stimuli (Prat et al., 2012), a maximum of 240 (Samur et al., 2015) and an average of 72 figurative stimuli. However, these numbers have to be regarded in context to the nature of the stimuli used.

The stimuli vary strongly in their structure and composition; with a span between single words and entire short stories (see Supplementary Table S5). Four studies used single words (Rüschemeyer et al., 2007; Forgács et al., 2012; Ma et al., 2016; Li et al., 2019). Word pairs (*n* = 15), or word pairs followed by a probe word (Forgács et al., 2015; Forgács, 2020), were chosen slightly more often, while two studies examined word triplets (Lee and Dapretto, 2006; Wang et al., 2021). Six studies tested with single phrases, an additional three combined their stimuli phrases with a prime, probe or target word. The majority of studies chose to work with sentences (*n* = 66), a further seven added a prime, probe or target word to their stimuli sentences. Five studies used sentence pairs (Bambini et al., 2011, 2016; Diaz and Hogstrom, 2011; Lai and Curran, 2013; Romero Lauro et al., 2013). A final eight studies (Eviatar and Just, 2006; Prat et al., 2012; Samur et al., 2015; Citron et al., 2016b; Hartung et al., 2020; Adamczyk et al., 2021; Deckert et al., 2021) worked with entire short stories. Consequently, 28 (24%) studies tested with isolated expressions while the remaining 76% presented context in varying degrees.

The figurative, i.e., critical, expressions can occur at different positions within a given stimulus. 20 papers do not specify any stimulus positions or allow for insight through example stimuli. One study (Forgács et al., 2015) continuously placed the critical expressions at the beginning of the stimuli, ten studies placed them in the middle, and 50 included them at the end. In 18 studies, the critical expressions varied in their positions within all stimuli. Among these, two exceptions can be found: De Grauwe et al. (2010) modulated the position of critical expressions within their stimuli in separate experiments, and Cardillo et al. (2012) contrasted stimuli with central and final positions of critical expressions. Note that this analysis was not applicable to 17 studies as the respective stimuli consisted entirely of figurative expressions.

The parts of speech of the figurative expressions are subject to variety, as well. 28 papers again either make no statement in this regard or do not allow for transparency by including sample stimuli. 28 studies worked exclusively with figurative nouns, nine studies included figurative verbs only. Two studies examined figurative adjectives specifically. The majority of studies (*n* = 49) however use a mixture of several PoS. Among those, four studies worked with either noun-noun pairs or noun-adjective pairs (Subramaniam et al., 2012, 2013; Zeev-Wolf et al., 2015; Forgács, 2020). Two papers (Benedek et al., 2014; Beaty et al., 2017) also describe exceptional cases as they had participants produce metaphors verbally, which makes a judgment of pre-determined PoS impossible.

Given the variety in the nature of the stimuli, it only follows that their lengths encompass a wide range as well. 52 (=44.8%) papers specify no stimuli lengths at all. The remaining papers differ in the units they use to state stimuli lengths. 40 papers make statements using the number of words, 14 papers use the number of letters. Four describe the length in syllables, three in the overall number of sentences per stimulus. One paper notes the number of phonemes. Three studies working with auditory stimuli give the number of milliseconds of their stimuli recordings. Studies with stimuli in Chinese state the stimuli length in number of characters (*n* = 9). Based on the respective length specifications, we calculated maximum, minimum and average numbers (see Supplementary Table S6).

#### Stimuli Sources and Rating Procedures

Not every set of stimuli was designed and controlled from scratch by the researchers using them. 73 studies state to have created their own stimulus material (no statement in this regard was interpreted as the authors having created the stimuli themselves, as well). Among those, 48 studies worked with their own material exclusively. The remaining 24 supplemented their own stimuli with material from external sources. 43 studies worked with material from outside sources only. Of those, 29 re-used stimuli from other studies. Four out of those 29 translated the original stimuli (Ibáñez et al., 2010, 2011; Beaty et al., 2017; Jończyk et al., 2020). The remaining studies used sources such as databases, dictionaries, or poetry.

In studies where stimuli were not obtained from external sources, stimulus control usually included rating procedures of various influence factors. The disagreement in working definitions as well as the frequent lack of definitions in the first place, as described in Definitions of Figurative Language section and Psycholinguistic Influence Factors section, leads to variation in basic operationalisation. Across all studies, these differences manifest in the methodology of attaining variable scores. Three practises for scoring are used primarily: ratings by a larger group of participants (“collective ratings”), judgments made by individuals (“expert ratings”), and the extraction of relevant data from databases.

Where collective ratings are employed, the rating procedures differ in respect to the number of participants and the rating scales. The stimuli are usually rated using scales of varying size: scales may present a binary choice (e.g., Kircher et al., 2007; Rüschemeyer et al., 2007; Lai et al., 2009; Bambini et al., 2016; Canal et al., 2017), more common are scales ranging from three to seven points. Few studies (*n* = 4) explicitly use expert ratings (Rüschemeyer et al., 2007; Lai et al., 2009; Forgács et al., 2012; Deckert et al., 2021).

Depending on the respective stimuli language, available databases provide data for several control factors, e.g., the MRC Psycholinguistic database (English; Coltheart, 1981) and CELEX (English, German, and Dutch; Baayen et al., 1995); these databases however do not distinguish between literal and figurative language. In contrast, newer databases as mentioned in section Structural Influence Factors provide data on figurative stimuli, but are used less often (Cardillo et al., 2010; Citron et al., 2016a, 2020a). Studies also make use of corpora: corpora are collections of natural language while linguistic databases are collections of single standardised linguistic entries. Objectively, corpora serve as a basis for calculating the frequency of occurrence as well as the cloze probability of relevant expressions.

The aspect of frequency allows for an analysis of operational variety. From a superficial perspective, frequency is controlled for often (59.5% of studies). However, studies arrive at their frequency scores in a variety of ways: some have frequency judged by subjective survey (Mashal et al., 2005, 2013, 2014; Goldstein et al., 2012; Lai et al., 2019), while others make use of corpus statistics (Proverbio et al., 2009; Yang et al., 2009; Rutter et al., 2012; Romero Lauro et al., 2013; Joue et al., 2020). Boulenger et al. (2009) and Uchiyama et al. (2012) constitute notable cases, as they refer to search results from Google (google.com) as a “corpus” without specifying if they applied any mechanisms to decrease the uncertainties in regard to language, site sources, register, site linkage, and repetition, and Forgács (2020) who determined the frequency of their stimuli by Google searches. The majority of studies work with pre-existing databases for their frequency scores. The database most cited (*n* = 9) is the MRC psycholinguistic database (Coltheart, 1981).

### Participants

Regarding participants' characteristics, we analysed age, handedness, gender, basic background, clinical status, and languages.

As illustrated in Supplementary Figure S4, participants mainly were 20–30 years of age and few studies included people over the age of 40 (*n* = 16). Participants were almost exclusively right-handed (*n* = 105, no statement: *n* = 6). We did not discover any significant imbalances in the gender ratios of participants. Overall, 2,712 people participated in the experiments described in our review corpus. Eight studies did not specify their participants' gender; of the remaining participants, 49.3% were male and 50.7 female. One paper (Hartung et al., 2020) mentions a non-binary person as part of the participant group. In all, there were seven studies which tested with male participants only (Bottini et al., 1994; Stringaris et al., 2006, 2007; Ahrens et al., 2007; Rüschemeyer et al., 2007; Kircher et al., 2009; Straube et al., 2011) and none that tested with female participants exclusively.

The participants predominantly consisted of university students or highly educated persons, as stated explicitly by 61 papers. 37 papers made no statement as to characteristics beyond age and gender of their participants. The remaining papers mention psychiatric diagnoses (clinical studies, *n* = 12) or refer to other factors (*n* = 8). The majority of papers report on research conducted with psychologically healthy adults (*n* = 104). The remaining studies examined patients with autism spectrum disorder (Chouinard et al., 2017; Kim et al., 2018), with schizophrenia (Iakimova et al., 2005; Kircher et al., 2007; Mashal et al., 2013, 2014; Straube et al., 2013, 2014; Zeev-Wolf et al., 2015; Adamczyk et al., 2021), with traumatic brain injury (Yang et al., 2010), and children with developmental language disorders and non-verbal learning disabilities (Lorusso et al., 2015).

As a consequence of our inclusion criteria, all papers were published in English. However, 59.5% of the studies were conducted with stimuli in languages other than English (see Table 3).

**Table 3:** Languages of the stimuli used in the studies (*n* = 116) in the review corpus.

**Language of stimuli**	***n***	**Language of stimuli**	***n***
English	44	Japanese	3
German	21	Spanish	2
Hebrew	11	Korean	1
Chinese	10	Norwegian	1
Italian	8	Polish	1
French	6	Russian	1
Dutch	4	N.A.	3

Three papers (Benedek et al., 2014; Beaty et al., 2017; Ojha et al., 2019) do not explicitly state which language their stimuli are in or contain ambiguous statements. Participants were predominantly native speakers of the respective stimuli languages (*n* = 100). Three studies examined L2-learners (Ibáñez et al., 2010; Ojha et al., 2019; Citron et al., 2020b), and 13 papers made no statement regarding this aspect.

## Discussion

In the discussion, we will focus on the following key aspects: definitions of figurative language, stimulus design with regard to the various aspects presented in the results section, and participants.

### Definitions of Figurative Language

The ratio of studied subtypes of figurative language in neuro-measurement studies marks a clear focus on metaphor. The processing of idioms on a neural level can therefore be considered less researched. Furthermore, there are hardly any studies deliberately contrasting figurative subtypes, making any potential processing differences virtually unknown.

The prevalent lack of definitions of figurative language and its subtypes indicates a shortfall in fundamental reflection of (cognitive-)linguistic theory in neuro-scientific research, resulting in ambiguous terminology across the scientific field. The lack of agreement on theoretical definitions makes comparisons between subtypes nearly impossible, indicating an even stronger need for mindful consideration of linguistic theory in studies that aim to examine one subtype only, in order to avoid accidental subtype overlap. It also clearly draws attention to the urgent need of working toward a consensus in theoretical foundations, especially in fundamental definitions, in order to develop a solid standard for empirical research to be based upon.

### Stimulus Design

#### Psycholinguistic Influence Factors

Overall, studies controlled for a high number of influence factors. The control for those factors is however unevenly distributed. The psycholinguistic influence factor most often controlled for is familiarity. The definitions of “familiarity” and “conventionality” illustrate the problem of inconsistent terminology and irregular operationalisation across studies. This problem is again not limited to these two terms; we will use them as the most prevalent example exemplifying the issue.

As described above, familiarity acts as a crucial influence on neural processing of figurative expressions: in their review, Bohrn et al. (2012) report stable activation in the right IFG and in the right anterior cingulate cortex (ACC) for novel metaphors but not for conventional/familiar ones (the two factors are not distinguished in this study); Schmidt and Seger (2009) affirm this by noting that, in their review, “all studies which report right hemisphere activation used novel or unfamiliar metaphors […] while most studies not reporting right hemisphere involvement […] do not use novel metaphors.” Kasparian (2013) succinctly summarises this by stating that not figurativeness, but instead familiarity modulates hemisphere involvement. This right lateralisation would be consistent with the GSH and indirectly with the CSCT, as well. However, since these reviews are largely based on the same studies the present review analyses, their results are affected by the same inherent inconsistencies in definitions.

We found discrepancies in definitions not only across studies, but also within studies as we compared theoretical and operational definitions. The aspect of continuum definitions and their actual experimental implementation showed that, taking familiarity as an example, factors defined as continuums are often operationalised binarily. A solution for this discrepancy is presented by the studies by Subramaniam et al. (2012); Romero Lauro et al. (2013); Zeev-Wolf et al. (2015), and Adamczyk et al. (2021): they established distinct ranges for central values on their scales, creating room for non-extreme values and therefore distinguishing the two ends of the scales more clearly. Citron et al. (2020b) also found a possibility to implement a continuum definition as such by including metaphoricity as a continuous variable in a parametric design in their final analysis. Cardillo et al. (2012) introduce a special case: they conducted a study where each individual participant's familiarity with metaphorical stimuli was continuously manipulated during the experiment, thereby circumventing collective rating scores entirely and soundly following the subjective-individual definition of familiarity.

Among the factors considered less often are concreteness, imageability, plausibility, and arousal. Conceptual concreteness and imageability influence memory periods and processing speed (Coltheart, 1981; Parker and Dagnall, 2009). Additionally, Citron et al. (2016b) describe a positive correlation between arousal and concreteness. Considering that conceptual metaphors enable the accessibility of abstract concepts through concrete domains, the spectrum of abstractness/concreteness gains an even more prominent role. Additionally, differences in figurative and literal plausibility can decide over processing demands—if an expression is literally implausible, the effort for judging between literal and figurative interpretation decreases (Bohrn et al., 2012; Kasparian, 2013).

We observed that only very few of the 116 papers define all of the factors that they controlled for, that definitions are remarkably rare in articles, and that they vary strongly across studies. This presents a problem for comparability: there are so many factors known to influence figurative language processing that it is virtually impossible to control for all of them. In turn, this means that comparing the results of studies which each controlled for different influence factors turns into a never-ending task of nestling apart the possible effects of each factor; a tedious and virtually impossible task as many factors influence each other and therefore represent confounding aspects. In consequence, imprecise terminology impedes a synthesis and comparison of neuroscientific research results.

#### Structural Stimulus Design

Given the great variation in composition, associated length, the examined parts of speech, and the position of figurative expressions within a stimulus, the stimuli are characterised by varying degrees of structural complexity. Contrast, for example, stimuli consisting of single metaphorical words such as “Stuhlbein” (chair leg, Forgács et al., 2012) and “begreifen” (grasp, to understand, Rüschemeyer et al., 2007) with the multi-sentence short stories used by Eviatar and Just (2006); Prat et al. (2012); Uchiyama et al. (2012); Samur et al. (2015); Citron et al. (2016b), and Hartung et al. (2020). The frequently used pattern of “A is B” hides a range in complexity, as well: Shibata et al. (2007) tested with stimuli such as “Difficulty is a wall,” while Bottini et al. (1994) represent the opposite end of the spectrum with sentences such as “The man who won the pools was a dog with the biggest bone.”

High syntactic complexity has been found to increase activation in the right hemisphere (Just et al., 1996; Constable et al., 2004; cf. Thoma and Daum, 2006) and generally requires more cognitive resources (Bohrn et al., 2012). This aspect consequentially adds to the factors influencing hemisphere recruitment; 46.6% of studies not controlling for syntactic complexity therefore risked confounding their results correspondingly. Additionally, processing verbs and nouns uses different neural systems (Damasio and Tranel, 1993) but the figurative expressions' part of speech is controlled for by only 44.8% of studies—about half of all studies therefore eliminate this confounding factor.

Furthermore, the overall nature of stimuli affects the predictability and context provided for an expression. According to the Configuration Hypothesis by Cacciari and Tabossi (1988), a speaker does not know whether they are processing an idiomatic or a literal expression until they recognise a familiar idiom in the sequence of single constituents. In other words, it is the predictability of an expression that determines its processing as an idiom, not the potential recall as a lexical unit. Later positions of figurative expressions in a stimulus therefore provide more context as support for disambiguation and predictability. Studies putting figurative expressions in an initial, central or in varying positions may therefore arrive at results that differ from studies with stimulus-final positions (Petten and Kutas, 1990; Canal et al., 2017). This fundamental (non-)existence and the degree of context influence the predictability and disambiguation processes, in turn influencing hemisphere recruitment (Diaz and Eppes, 2018).

The statements of length and position of critical elements present an avoidable obstacle for information synthesis: frequently, papers lack information on these two aspects; the position of critical expressions cannot always be reliably extracted from given stimuli examples. The results yield non-representative numbers which negate any reliable comparability in this aspect.

#### Stimuli Sources and Rating Procedures

Part of the studies worked with stimulus material taken from earlier studies. On the one hand, this is an important factor for assessing the replicability and validity of those studies. On the other hand, stimuli recycling has to be considered in a comparison of research results in order to avoid an influence of a particular stimulus set. However, we did not find any stimulus sets that were used frequently enough to raise concerns about a bias in this respect.

The aspect of frequency illustrated the broad variability in the implementation of rating procedures. This demonstrates that, even if a term is used to name a certain factor in several studies, it may encompass several operational differences and therefore variation in the scores attained. These scores may carry inherent assumptions: a study implementing frequency as subjective frequency stands in stark contrast to a study collecting Google results, which in turn will have different results than a study taking its scores from an already established database.

As we observed, the most popular database in our review corpus is the MRC database. However, the data in this database are a collection of smaller studies and databases originating from 1944 to 1986. This gives reason to assume that the scores of some words for variables such as frequency (cf. Brysbaert and New, 2009) and familiarity might have changed in the meantime. In general, databases most importantly contain an inherent problem for figurative language research: the frequency of metaphorical meanings cannot be objectively extracted from databases and corpora. Instead, frequency scores are taken for content words or the single critical words only (Sotillo et al., 2005; Boulenger et al., 2009; Raposo et al., 2009; Yang et al., 2009, 2013; Diaz and Hogstrom, 2011; Rommers et al., 2013; Jończyk et al., 2020; Yurchenko et al., 2020). The frequency scores therefore cannot be interpreted as the frequency of metaphors *per se*, but as the frequency of their respective lexical units independent of literal or figurative meaning. An interpretation as anything other than frequency of lexical occurrence must consequently be viewed with severe caution.

### Participants

The studies in our review corpus show a clear bias toward highly educated, young adult participants. Participants were mostly university students, which is a common and difficult to avoid phenomenon: since most neurolinguistic research is done at universities, university students represent the easiest to obtain and ever-changing pool of potential participants. This bias is therefore not to be interpreted as an oversight but as a pragmatic consequence of academic circumstance. However, this does limit the representativeness of results for the general population. Mejía-Constaín et al. (2010) report age as an influential factor on figurative language processing, and the level of (higher) education has been proven to interact with general language abilities and reading levels (cf. Levine et al., 2020). Almost all studies successfully balanced gender in their participant groups, and recent studies are taking steps away from the gender binary by noting non-binary participants. Since there were only 12 studies with clinical populations, and within those mainly studies on schizophrenia, future reseach would benefit especially from more studies on the processing of figurative language by diverse clinical groups.

Regarding the languages examined in our review corpus, we found a clear bias in the stimulus language distribution: the stimulus material in 74.1% of studies is composed in either a Romance (13.8%) or a Germanic language (60.3%), with English accounting for 37.9% of all stimuli languages. The remaining parts are represented by Hebrew, several distinct languages from the Asian language area (Chinese, Japanese, Korean), and Slavic languages (Polish, Russian). However, our results carry a bias inherent to the authors' language proficiencies and the inclusion criteria of English as a publication language. We cannot make any statements regarding language distributions in studies published in languages other than English.

However, the described bias in papers published in English introduces the danger of overgeneralising research results based on stimulus material in specific European languages onto other language groups. Considering syntactic, semantic, and morphological cross-linguistic differences and the potential cultural character of metaphors (Kövecses, 2010), such a generalisation would be inappropriate. A future synthesis of studies published in languages other than English would therefore be beneficial.

## Conclusion and Outlook

Our review identified fundamental similarities and differences in the methodology of neurolinguistic studies on metaphor and idiom processing. On the one hand, varied stimulus design provides a diverse basis for research. On the other hand, the theoretical and operational differences critically impede the general comparability of results, and therefore result in the need for careful and detailed consideration of applied methods.

As proposed by pre-existing literature (e.g., Kasparian, 2013; Wang and He, 2013; Diaz and Eppes, 2018), certain influence factors known to influence lateralisation (e.g., syntactic complexity, familiarity, task demand, context, or predictability) might affect the hemispheric activation during the processing of figurative language. Hemispheric differences are therefore indicated to stem from the influence of psycholinguistic and structural influence factors and not the contrast between figurativeness and literalness *per se*. It is therefore crucial to control for said influence factors in a conscientious and transparent manner.

In realistic terms, it would be impossible to control for every known influence factor. Consequently, our review does not conclude in a demand for unreasonably extensive stimulus control. Our overarching aim is encouraging more transparent research, both in practise and in publication, in order to enable better comparability and replicability. This could be achieved by defining all terms and parameters in clear, unambiguous language. For this, it may be beneficial to consider previous research and examine the terms and operationalisation thereof carefully in order to avoid unfortunate overlap in terminology and in order to avoid the synthesis of results based on dissimilar methodology. Additionally, publishing the stimulus material would increase research transparency and help other researcher replicate and validate previous results. In line with this, publishing research (including text, figures, tables, data, and any other additional Supplementary Material) could make the research accessible for the widest possible audience.

On a concluding note, our review was limited in several aspects. As mentioned, we only included papers published in English. The analysis of stimulus control (and possibly results) of studies in languages other than English would therefore be a beneficial future endeavour. We also did not include data concerning presentation, task nature and difficulty (cf. Schmidt and Seger, 2009), operational contrasts, analysis methods, and results for our review of research methods. A future review with our literature corpus examining those aspects would complement the present review, further driving insights on how to compare and design neurolinguistic studies conscientiously.

## Data Availability Statement

The datasets presented in this study can be found in online repositories. The names of the repository/repositories and accession number(s) can be found in the article/Supplementary Material.

## Author Contributions

SK: literature synthesis, data analysis, data interpretation, manuscript drafting, writing and editing, and creation of figures and tables. NM: manuscript writing and editing and contribution to data interpretation. CK: conceptualisation, supervision, manuscript editing, and contribution to manuscript writing. All authors contributed to the article and approved the submitted version.

## Funding

The publication of this research was funded by the Deutsche Forschungsgesellschaft, award number KA 2258/2-1.

## Conflict of Interest

The authors declare that the research was conducted in the absence of any commercial or financial relationships that could be construed as a potential conflict of interest.

## Publisher's Note

All claims expressed in this article are solely those of the authors and do not necessarily represent those of their affiliated organizations, or those of the publisher, the editors and the reviewers. Any product that may be evaluated in this article, or claim that may be made by its manufacturer, is not guaranteed or endorsed by the publisher.

## Footnotes

^1^All figures and tables beginning with “S” can be found in the Supplementary Material.

^2^Which excludes titles such as “Using the Brain as a Metaphor to Model Flexible Production Systems” (Garud and Kotha, 1994).
