# Supplementary material for: The Elephant in the Room: A Systematic Review of Stimulus Control in Neuro-Measurement Studies on Figurative Language Processing
Source: Front Hum Neurosci. 2022 Jan 21;15:791374. doi: 10.3389/fnhum.2021.791374 (PMC8814624; doi:10.3389/fnhum.2021.791374)
Supplement: Supplementary file 2 [file Data_Sheet_2.pdf]

Supplementary Material B

Table S3: Overview of the 116 research articles in the review corpus.

Author(s)	Title	Year	Measuring Method	Figurative mean	Language Stimuli
Ahrens et al.	Functional MRI of conventional and anomalous metaphors in Mandarin Chinese	2007	fMRI	Metaphor	Chinese (Mandarin)
Adamczyk et al.	On the role of bilateral brain hypofunction and abnormal lateralization of cortical information flow as neural underpinnings of conventional metaphor processing impairment in schizophrenia: an fMRI and EEG study	2021	fMRI & EEG	Metaphor	Polish
Arzouan et al.	Brainwaves are stethoscopes: ERP correlates of novel metaphor comprehension	2007	EEG	Metaphor	Hebrew
Arzouan et al.	Dynamics of hemispheric activity during metaphor comprehension: electrophysiological measures	2007	EEG	Metaphor	Hebrew
Arzouan et al.	Big words, halved brains and small worlds: complex brain networks of figurative language comprehension	2011	EEG	Metaphor	Hebrew
Aziz-Zadeh et al.	Congruent embodied representations for visually presented actions and linguistic phrases describing actions	2006	fMRI	Metaphor & Idiom	English
Bambini et al.	Decomposing metaphor processing at the cognitive and neural level through functional magnetic resonance imaging	2011	fMRI	Metaphor	Italian
Bambini et al.	Disentangling metaphor from context: an ERP study	2016	EEG	Metaphor	Italian
Beaty et al.	Brain networks underlying novel metaphor production	2017	fMRI	Metaphor	NA (likely English, otherwise German)
Benedek et al.	Creating metaphors: the neural basis of figurative language production	2014	fMRI	Metaphor	NA (likely German, otherwise English)
Bottini et al.	The role of the right-hemisphere in the interpretation of figurative aspects of language - a positron emission tomography activation study	1994	PET	Metaphor	English
Boulenger et al.	Grasping ideas with the motor system: semantic somatotopy in idiom comprehension	2009	fMRI	Idiom	English
Boulenger et al.	When do you grasp the idea? MEG evidence for instantaneous idiom understanding	2012	MEG	Idiom	English
Canal et al.	Basic composition and enriched integration in idiom processing: an EEG study	2017	EEG	Idiom	Italian

Author(s)	Title	Year	Measuring Method	Figurative mean	Language Stimuli
Cardillo et al.	From novel to familiar: tuning the brain for metaphors	2012	fMRI	Metaphor	English
Chen et al.	Functional-anatomical organization of predicate metaphor processing	2008	fMRI	Metaphor	English
Chouinard et al.	Neurological evaluation of the selection stage of metaphor comprehension in individuals with and without autism spectrum disorder	2017	fMRI	Metaphor	English
Citron et al.	Conventional metaphors in longer passages evoke affective brain response	2016	fMRI	Metaphor	German
Citron et al.	Metaphorical language processing and amygdala activation in L1 and L2	2020	fMRI	Metaphor	German
Citron et al.	Idiomatic expressions evoke stronger emotional responses in the brain than literal sentences	2019	fMRI	Idiom	German
Coulson & Van Petten	Conceptual integration and metaphor: an event-related potential study	2002	EEG	Metaphor	English
Coulson & Van Petten	A special role for the right hemisphere in metaphor comprehension? ERP evidence from hemifield presentation	2007	EEG	Metaphor	English
Couto et al.	Effective connectivity study guiding the neuromodulation intervention in figurative language comprehension using optical neuroimaging	2020	fNIRS	Metaphor	Chinese
Deckert et al.	Electrophysiological correlates of conventional metaphor, irony, and literal language processing? An event-related potentials and eLORETA study	2021	EEG	Metaphor & Irony	German
De Grauwe et al.	Electrophysiological insights into the processing of nominal metaphors	2010	EEG	Metaphor	English
Desai et al.	The neural career of sensory-motor metaphors	2011	fMRI	Metaphor	English
Desai et al.	A piece of the action: modulation of sensory-motor regions by action idioms and metaphors	2013	fMRI	Metaphor & Idiom	English
Diaz & Hogstrom	The influence of context on hemispheric recruitment during metaphor processing	2011	fMRI	Metaphor	English
Diaz et al.	The influence of sentence novelty and figurativeness on brain activity	2011	fMRI	Metaphor	English
Eviatar & Just	Brain correlates of discourse processing: an fMRI investigation of irony and conventional metaphor comprehension	2006	fMRI	Metaphor & Irony	English
Fondevila et al.	Counterintuitive religious ideas and metaphoric thinking: an event-related brain potential study	2016	EEG	Metaphor	Spanish
Forgács et al.	Neural correlates of combinatorial semantic processing of literal and figurative noun noun compound words	2012	fMRI	Metaphor	German

Author(s)	Title	Year	Measuring Method	Figurative mean	Language Stimuli
Forgács et al.	Metaphors are physical and abstract: ERPs to metaphorically modified nouns resemble ERPs to abstract language	2015	EEG	Metaphor	English
Forgács	An electrophysiological abstractness effect for metaphorical meaning making	2020	EEG	Metaphor	French
Goldstein et al.	Killing a novel metaphor and reviving a dead one: ERP correlates of metaphor conventionalization	2012	EEG	Metaphor	Hebrew
Hartung et al.	Context matters: novel metaphors in supportive and non-supportive contexts	2020	fMRI	Metaphor	English
Hillert & Buračas	The neural substrates of spoken idiom comprehension	2009	fMRI	Idiom	English
Iakimova et al.	ERPs of metaphoric, literal, and incongruous semantic processing in schizophrenia	2005	EEG	Metaphor	French
Ibáñez et al.	Gesture influences the processing of figurative language in non-native speakers: ERP evidence	2010	EEG	Metaphor	German
Ibáñez et al.	High contextual sensitivity of metaphorical expressions and gesture blending: a video event-related potential design	2011	EEG	Metaphor	German
Ji et al.	The role of animacy in metaphor processing of Mandarin Chinese: an Event-Related Potential (ERP) study	2020	EEG	Metaphor	Chinese
Jonczyk et al.	Engineering creativity: prior experience modulates electrophysiological responses to novel metaphors	2020	EEG	Metaphor	English
Joue et al.	Metaphor processing is supramodal semantic processing: the role of the bilateral lateral temporal regions in multimodal communication	2020	fMRI	Metaphor	German
Kazmerski et al.	ERP and behavioral evidence of individual differences in metaphor comprehension	2003	EEG	Metaphor	English
Kessler et al.	Activation of literal word meanings in idioms: evidence from eye-tracking and ERP experiments	2020	EEG	Idiom	German
Kim et al.	Aberrant neural activation underlying idiom comprehension in korean children with high functioning autism spectrum disorder	2018	fMRI	Idiom	Korean
Kircher et al.	Neural correlates of metaphor processing in schizophrenia	2007	fMRI	Metaphor	German
Kircher et al.	Neural interaction of speech and gesture: differential activations of metaphoric co-verbal gestures	2009	fMRI	Metaphor	German

Author(s)	Title	Year	Measuring Method	Figurative mean	Language Stimuli
Klepousniotou et al.	Pathways to lexical ambiguity: fMRI evidence for bilateral fronto-parietal involvement in language processing	2014	fMRI	Metaphor	English
Lacey et al.	Metaphorically feeling: comprehending textural metaphors activates somatosensory cortex	2012	fMRI	Metaphor	English
Lacey et al.	Engagement of the left extrastriate body area during body-part metaphor comprehension	2017	fMRI	Metaphor	English
Lachaud	Conceptual metaphors and embodied cognition: EEG coherence reveals brain activity differences between primary and complex conceptual metaphors during comprehension	2013	EEG	Metaphor	Norwegian
Lai & Curran	ERP evidence for conceptual mappings and comparison processes during the comprehension of conventional and novel metaphors	2013	EEG	Metaphor & Simile	English
Lai & Desai	The grounding of temporal metaphors	2016	fMRI	Metaphor	English
Lai et al.	Comprehending conventional and novel metaphors: an ERP study	2009	EEG	Metaphor	English
Lai et al.	Familiarity differentially affects right hemisphere contributions to processing metaphors and literals	2015	fMRI	Metaphor	English
Lai et al.	Concrete processing of action metaphors: evidence from ERP	2019	EEG	Metaphor	English
Laurent et al.	On understanding idiomatic language: the salience hypothesis assessed by ERPs	2006	EEG	Idiom	French
Lauro et al.	Idiom comprehension: a prefrontal task?	2008	fMRI	Idiom	Italian
Lee & Dapretto	Metaphorical vs. literal word meanings: fMRI evidence against a selective role of the right hemisphere	2006	fMRI	Metaphor	English
Lorusso et al.	Processing sentences with literal versus figurative use of verbs: an ERP study with children with language impairments, nonverbal impairments, and typical development	2015	EEG	Metaphor & Idiom	Italian
Lu & Zhang	Event-related potential evidence for the early activation of literal meaning during comprehension of conventional lexical metaphors	2012	EEG	Metaphor	Chinese (Mandarin)
Ma et al.	Neural correlates of multimodal metaphor comprehension: evidence from event-related potentials and time-frequency decompositions	2016	EEG	Metaphor	Chinese
Mashal et al.	The role of the right hemisphere in processing nonsalient metaphorical meanings: application of Principal Components Analysis to fMRI data	2005	fMRI	Metaphor	Hebrew

Author(s)	Title	Year	Measuring Method	Figurative mean	Language Stimuli
Mashal et al.	An fMRI investigation of the neural correlates underlying the processing of novel metaphoric expressions	2007	fMRI	Metaphor	Hebrew
Mashal et al.	An fMRI study of processing novel metaphoric sentences	2009	fMRI	Metaphor	Hebrew
Mashal et al.	Enhanced left frontal involvement during novel metaphor comprehension in schizophrenia: evidence from functional neuroimaging	2013	fMRI	Metaphor	Hebrew
Mashal et al.	The role of the precuneus in metaphor comprehension: evidence from an fMRI study in people with schizophrenia and healthy participants	2014	fMRI	Metaphor	Hebrew
Mashal et al.	An fMRI investigation of the neural correlates underlying the processing of novel metaphoric expressions	2008	fMRI	Idiom	Hebrew
Mejía-Constaín et al.	When metaphors go literally beyond their territories: the impact of age on figurative language	2010	fMRI	Metaphor	French
Morid et al.	Capturing the multi-determined nature of idiom processing using ERPs	2021	EEG	Idiom	English
Obert et al.	Differential bilateral involvement of the parietal gyrus during predicative metaphor processing: an auditory fMRI study	2014	fMRI	Metaphor	French
Ojha et al.	Similarities and differences between verbal and visual metaphor processing: an EEG study	2019	EEG	Metaphor	NA (English or Japanese)
Pomp et al.	Lexical olfaction recruits olfactory orbitofrontal cortex in metaphorical and literal contexts	2018	fMRI	Metaphor	German
Prat et al.	An fMRI investigation of analogical mapping in metaphor comprehension: the influence of context and individual cognitive capacities on processing demands	2012	fMRI	Metaphor & Irony	English
Proverbio et al.	The role of left and right hemispheres in the comprehension of idiomatic language: an electrical neuroimaging study	2009	EEG	Idiom	Italian
Pynte et al.	The time-course of metaphor comprehension: an event-related potential study	1996	EEG	Metaphor	French
Raposo et al.	Modulation of motor and premotor cortices by actions, action words and action sentences	2009	fMRI	Idiom	English
Rapp et al.	Neural correlates of metaphor processing	2004	fMRI	Metaphor	German
Rapp et al.	Laterality in metaphor processing: lack of evidence from functional magnetic resonance imaging for the right hemisphere theory	2007	fMRI	Metaphor	German

Author(s)	Title	Year	Measuring Method	Figurative mean	Language Stimuli
Romero Lauro et al.	She runs, the road runs, my mind runs, bad blood runs between us: literal and figurative motion verbs: an fMRI study	2013	fMRI	Metaphor & Idiom	Italian
Rommers et al.	Context-dependent semantic processing in the human brain: evidence from idiom comprehension	2013	EEG	Idiom	Dutch
Rüschemeyer et al.	Comprehending prehending: Neural correlates of processing verbs with motor stems	2007	fMRI	Metaphor	German
Rutter et al.	Can clouds dance? Neural correlates of passive conceptual expansion using a metaphor processing task: Implications for creative cognition	2012	fMRI	Metaphor	German
Samur et al.	Emotional context modulates embodied metaphor comprehension	2015	fMRI	Metaphor	Dutch
Schmidt & Seger	Neural correlates of metaphor processing: The roles of figurativeness, familiarity and difficulty	2009	fMRI	Metaphor	English
Schmidt-Snoek et al.	Auditory and motion metaphors have different scalp distributions: an ERP study	2015	EEG	Metaphor	English
Schneider et al.	Beyond the N400: complementary access to early neural correlates of novel metaphor comprehension using combined electrophysiological and haemodynamic measurements	2014	EEG & NIRS	Metaphor	German
Schuil et al.	Sentential context modulates the involvement of the motor cortex in action language processing: an fMRI study	2013	fMRI	Metaphor & Idiom	Dutch
Shibata et al.	Neural mechanisms involved in the comprehension of metaphoric and literal sentences: an fMRI study	2007	fMRI	Metaphor	Japanese
Shibata et al.	Does simile comprehension differ from metaphor comprehension? A functional MRI study	2012	fMRI	Metaphor & Simile	Japanese
Solomon & Thompson-Schill	Finding features, figuratively	2017	fMRI	Metaphor	English
Sotillo et al.	Neural activity associated with metaphor comprehension: spatial analysis	2005	EEG	Metaphor	Spanish
Straube et al.	The differentiation of iconic and metaphoric gestures: common and unique integration processes	2011	fMRI	Metaphor	German
Straube et al.	Neural integration of speech and gesture in schizophrenia: evidence for differential processing of metaphoric gestures	2013	fMRI	Metaphor	German

Author(s)	Title	Year	Measuring Method	Figurative mean	Language Stimuli
Straube et al.	Superior temporal sulcus disconnectivity during processing of metaphoric gestures in schizophrenia	2014	fMRI	Metaphor	German
Stringaris et al.	How metaphors influence semantic relatedness judgments: the role of the right frontal cortex	2006	fMRI	Metaphor	English
Stringaris et al.	Deriving meaning: distinct neural mechanisms for metaphoric, literal, and non-meaningful sentences	2007	fMRI	Metaphor	English
Subramaniam et al.	The Repetition Paradigm: enhancement of novel metaphors and suppression of conventional metaphors in the left inferior parietal lobe	2012	fMRI	Metaphor	English
Subramaniam et al.	Positively valenced stimuli facilitate creative novel metaphoric processes by enhancing medial prefrontal cortical activation	2013	fMRI	Metaphor	English
Tang et al.	Comprehension of scientific metaphors: complementary processes revealed by ERP	2017	EEG	Metaphor	Chinese
Tang et al.	The temporal dynamics underlying the comprehension of scientific metaphors and poetic metaphors	2017	EEG	Metaphor	Chinese
Tartter et al.	Novel metaphors appear anomalous at least momentarily: evidence from N400	2002	EEG	Metaphor	English
Uchiyama et al.	Distinction between the literal and intended meanings of sentences: a functional magnetic resonance imaging study of metaphor and sarcasm	2012	fMRI	Metaphor & Sarcasm	Japanese
Vespignani et al.	Predictive mechanisms in idiom comprehension	2010	EEG	Idiom	Italian
Wang et al.	The roles of familiarity and context in processing Chinese xiehouyu: an ERP study	2021	EEG	Metaphor & Idiom	Chinese
Weiland et al.	The role of literal meaning in figurative language comprehension: evidence from masked priming ERP	2014	EEG	Metaphor & Metonymy	German
Yang et al.	Differences in task demands influence the hemispheric lateralization and neural correlates of metaphor	2009	fMRI	Metaphor	English
Yang et al.	Figurative language processing after traumatic brain injury in adults: a preliminary study	2010	fMRI	Metaphor	English
Yang et al.	Contextual effects on conceptual blending in metaphors: an event-related potential study	2013	EEG	Metaphor	English

Author(s)	Title	Year	Measuring Method	Figurative mean	Language Stimuli
Yurchenko et al.	Metaphor is between metonymy and homonymy: evidence from event-related potentials	2020	EEG	Metaphor & Metonymy	Russian
Zane & Shafer	Mixed metaphors: electrophysiological brain responses to (un)expected concrete and abstract prepositional phrases	2018	EEG	Metaphor & Idiom	English
Zeev-Wolf et al.	Magnetoencephalographic evidence of early right hemisphere overactivation during metaphor comprehension in schizophrenia	2015	MEG	Metaphor	Hebrew
Zempleni et al.	Evidence for bilateral involvement in idiom comprehension: an fMRI study	2007	fMRI	Idiom	Dutch
Zhang et al.	ERP correlates of compositionality in Chinese idiom comprehension	2013	EEG	Idiom	Chinese
Zhou et al.	Spatiotemporal analysis of ERP during chinese idiom comprehension	2004	EEG	Idiom	Chinese
