# Supplementary material for: The Elephant in the Room: A Systematic Review of Stimulus Control in Neuro-Measurement Studies on Figurative Language Processing
Source: Front Hum Neurosci. 2022 Jan 21;15:791374. doi: 10.3389/fnhum.2021.791374 (PMC8814624; doi:10.3389/fnhum.2021.791374)
Supplement: Supplementary file 3 [file Data_Sheet_3.pdf]

Supplementary Material C: Additional information

Contents

Table S1: Short overview over the figurative subtypes idiom, irony, sarcasm, metaphor, metonymy, and proverb……………………………………………………………………………………2

Search term and search limitations in the Web of Science database………………………………..3

Figure S1: Overview of the publication years of the scientific papers in the review corpus (n = 116). 4

Figure S2: Comparison of the neuro-measurement methods employed by the studies in the review corpus (n = 116).……………………………………………………………………….……………5

Table S4: Cognitive models of figurative language processing most frequently mentioned in the review corpus. Total papers in the review corpus: n = 116….……………………………………...6

Figure S3: Medium of the stimulus material. Note that all 116 research papers had to include figurative-linguistic stimuli and that employing additional media was optional……………………... 7

Figure S4: Participants' minimum (A), maximum (B) and average ages (C). Total studies n = 116. Papers making no statements regarding minimum age n = 45, regarding maximum age n = 45, regarding average age n = 15…………………………………...…………………………………... 8

Table S5: Summary of stimuli kinds and their respective numbers used by studies in the review corpus (n = 116), divided into total stimuli and figurative stimuli only………………………………9

Table S6: Summary of stimuli lengths given in the units of measurements stated by the respective papers. Note that the number of papers making no statement in this regard is particularly high, and that papers frequently give averages only, leading to at a first glance contradictory results…………. 10

Table S1: Short overview over the figurative subtypes idiom, irony, sarcasm, metaphor, metonymy, and proverb.

Subtype	Working definition	Examples
Idiom	Idioms are conventional multi-word expressions of rigid syntactic structures whose meaning cannot be extracted by the meaning of its single constituents.	• to kick the bucket<br>• to be on thin ice
Irony and Sarcasm	Irony and its more severe form, sarcasm, are not inherently figurative statements; instead, the literal meaning is "falsified by context information" (Thoma & Daum, 2006). The subtypes are therefore marked by an especially stark contrast between the meaning of the literal utterance and the actually intended meaning (Thoma & Daum, 2006; Bohrn et al., 2012) in spontaneous situational context. Pragmatic abilities have to be intact both for recognition and integration of context, and for the correct comprehension of the speaker's intentions. Both irony and sarcasm differ from other forms of figurative language in their exclusive creative emergence in spontaneous communication and consequent exemption from conventionalisation.	• Person A: (*tells a really bad joke)* Person B: "Wow, that was so funny..."<br><br>• Person A: (*falls down the stairs)* Person B: "That was graceful."
Metaphor	As our working definition, we define metaphors as free nonliteral expressions which are not subject to rigid structure and which follow the notion of conceptual metaphor after Lakoff and Johnson's CMT (1980), i.e. metaphors as a conceptualisation mechanism with a source and a target domain.	• This mom is a <u>lion</u>.<br>• She is feeling <u>bitter</u>.
Metonomy	Metonymy conceptualises elements of one concept by elements of the same concept, or a concept as a whole is conceptualized by one of its elements (Weiland et al., 2014; Canal et al., 2017) – in the terms of CMT, a metonymy performs mappings within one frame.	• The White House<br>• Table leg
Proverb	Proverbs are "familiar, fixed, sentential expressions that communicate well-known truths, social norms, or moral concerns" (Gibbs & Beitel, 1995: 134) and therefore differ from metaphor and idiom primarily in their high degree of polylexicality and their pragmatic function.	• Better safe than sorry<br>• Never change a running system

Search term and search limitations in the Web of Science database

Taking the WoS database as an example, the complete search term read as follows:

> TS=((("figurative language" OR non-literal OR proverb OR idiom OR metonymy OR simile OR sarcasm OR irony OR metaphor) AND (language OR comprehension OR processing OR linguistic)) AND (neurology OR neurophys OR imaging OR brain OR hemisphere OR fMRI OR EEG OR PET OR ERP OR MEG)) OR AB=((("figurative language" OR non-literal OR proverb OR idiom OR metonymy OR sarcasm OR irony OR metaphor) AND (language OR comprehension OR processing OR linguistic)) AND (neurology OR neurophys OR imaging OR brain OR hemisphere OR fMRI OR EEG OR PET OR ERP OR MEG)

In the case of WoS, the time window for publication date was entered via the graphic filter options. We deliberately abstained from limiting the search to certain fields of study (as would be possible via the MeSH terms in PubMed) in order to avoid excluding untagged sources. The precise search terms and additionally employed functions naturally varied slightly for the remaining three databases.

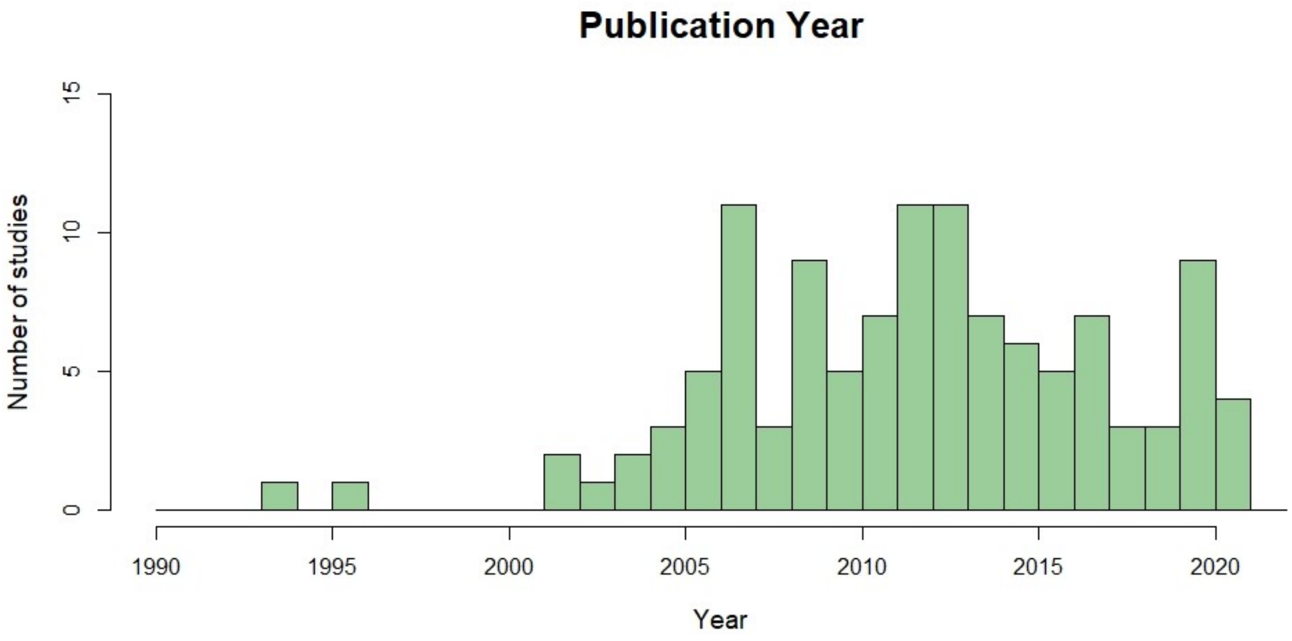

Figure S1: Overview of the publication years of the scientific papers in the review corpus (n = 116).

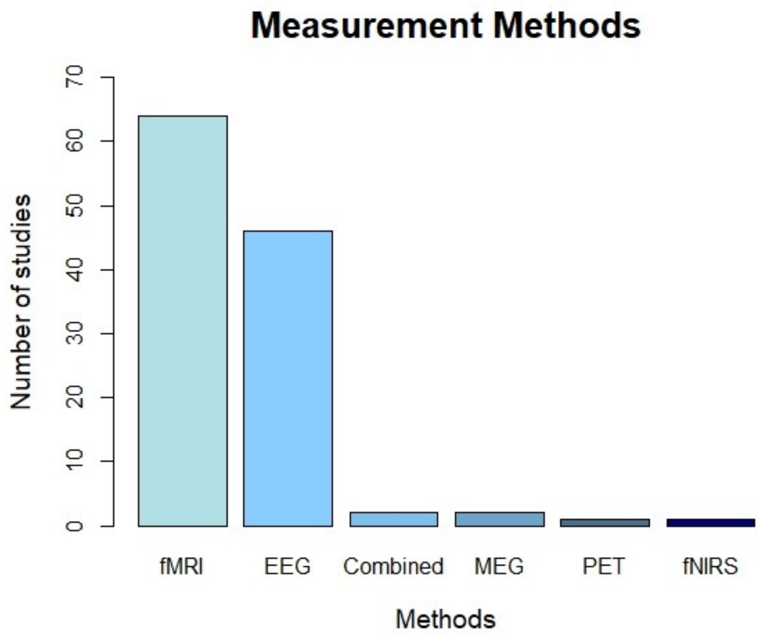

Figure S2: Comparison of the neuro-measurement methods employed by the studies in the review corpus (n = 116).

Table S4: Cognitive models of figurative language processing most frequently mentioned in the review corpus. Total papers in the review corpus: n = 116.

**Cognitive Model**	**Mentions (n)**
Conceptual Metaphor Theory (George Lakoff and Mark Johnson)	30
Graded Salience Hypothesis (Rachel Giora)	27
Coarse Semantic Coding Theory (Mark Beeman)	19
Structural Mapping Theory (Gentner, 1983)	9
Configuration Hypothesis (Cacciari & Tabossi, 1988)	8
Lexical Representation Hypothesis (Swinney & Cutler, 1979)	4
Conceptual Blending Theory (Turner & Fauconnier, 2002)	4

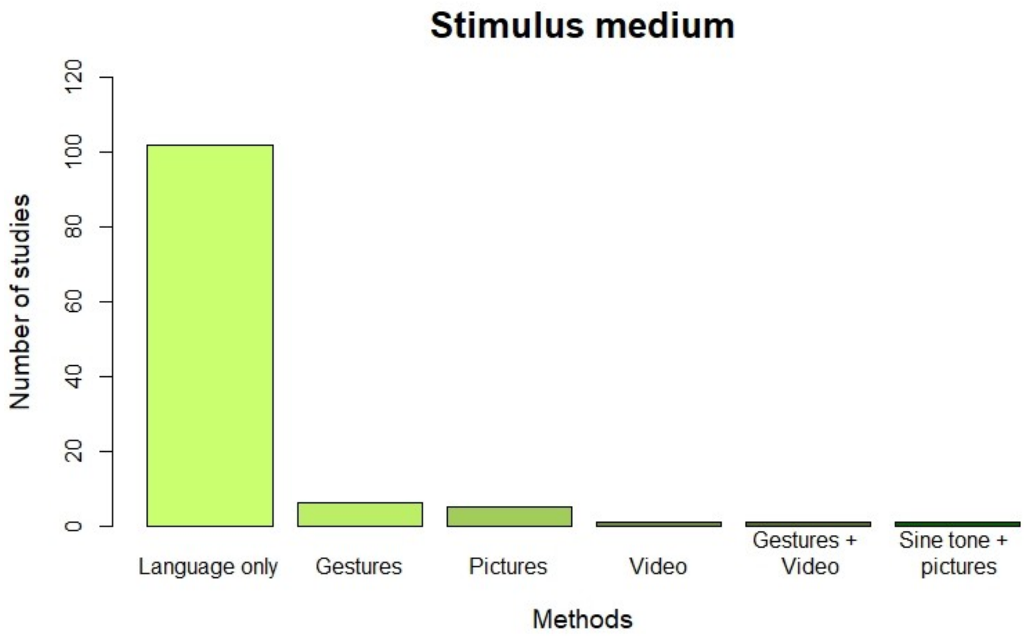

Figure S3: Medium of the stimulus material. Note that all 116 research papers had to include figurative-linguistic stimuli and that employing additional media was optional.

(A)

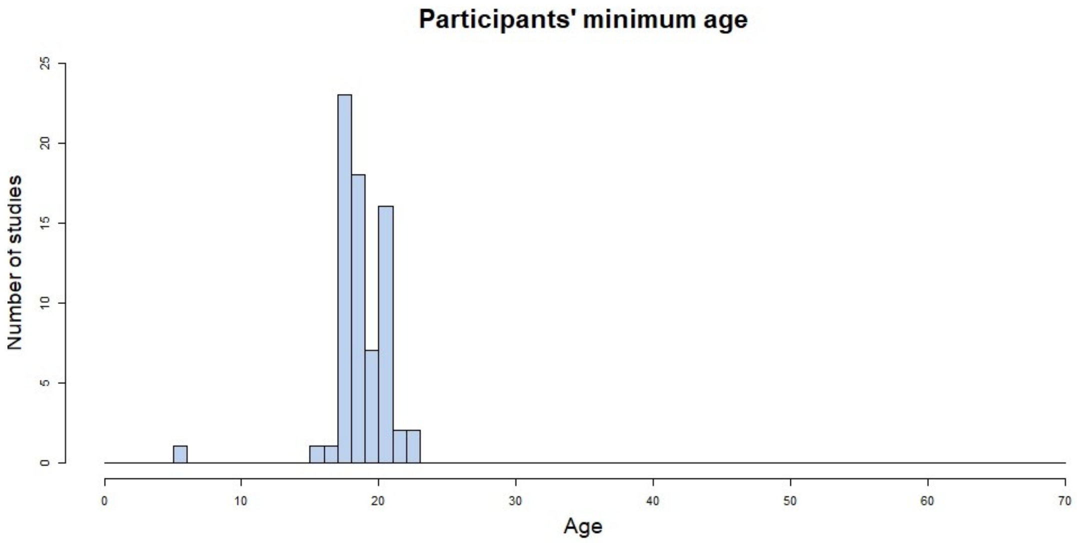

(B)

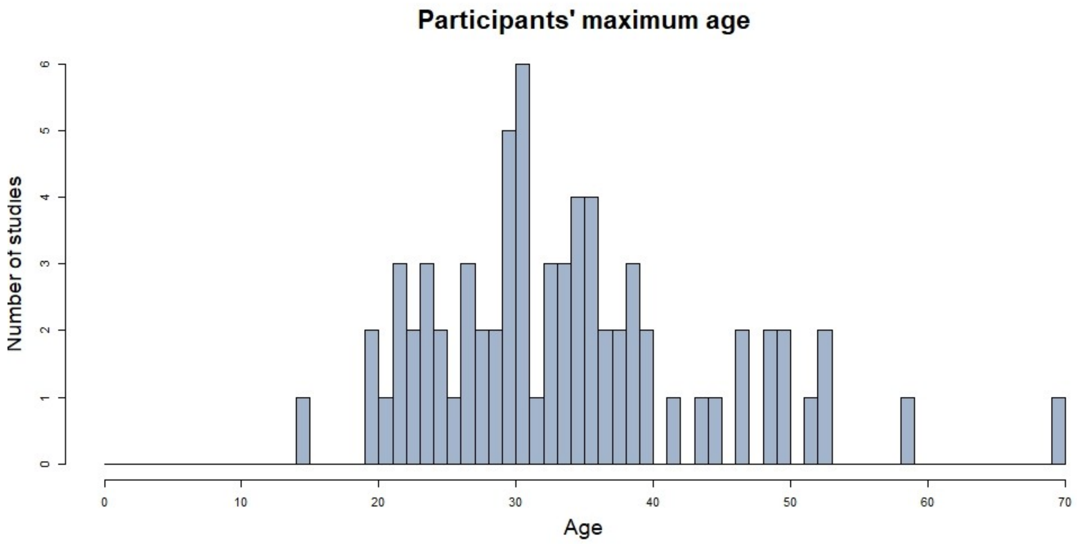

(C)

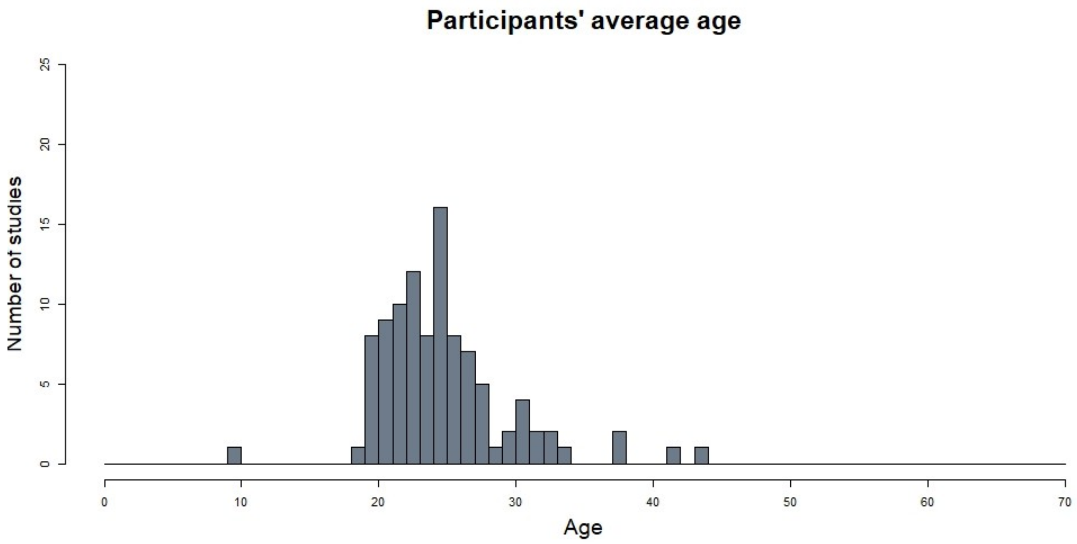

Figure S4: Participants' minimum (A), maximum (B) and average ages (C). Total studies n = 116. Papers making no statements regarding minimum age n = 45, regarding maximum age n = 45, regarding average age n = 15.

Table S5: Summary of stimuli kinds and their respective numbers used by studies in the review corpus (n = 116), divided into total stimuli and figurative stimuli only.

	Phrases	Phrases + word	Sentence pairs	Sentences	Sentences + word	Short stories	Word pairs	Word pairs + probe	Word triplets	Words	**All**
**All stimuli**											
**Min**	30	28	120	25	25	22	96	400	32	60	22
**Max**	144	240	384	1024	624	576	635	636	240	200	1024
**ø**	89.8	153.3	247	200.7	264.6	133.8	176.2	518	136	132.4	194.4
**Figurative stimuli**											
**Min**	15	28	40	12	40	10	25	105	16	30	10
**Max**	80	120	192	208	180	240	136	200	80	100	240
**ø**	44.6	85.3	83.6	69.2	103.2	55.6	76.8	152.5	48	70	72

Table S6: Summary of stimuli lengths given in the units of measurements stated by the respective papers. Note that the number of papers making no statement in this regard is particularly high, and that papers frequently give averages only, leading to at a first glance contradictory results.

**Unit of measurement**	**Minimum**	**Maximum**	**Average**	**Number of statements**
Letters	2	8	22.7	14
Chin. characters	1	12	4.9	9
Phonemes	NA	NA	28.61	1
Syllables	NA	NA	10.7	4
Words	2	100	6.6	40
Sentences	3	4	3.3	3
Milliseconds	1200	1800	3816	3
No statement	NA	NA	NA	52
